# Lack of association of *C3* gene with uveitis: additional insights into the genetic profile of uveitis regarding complement pathway genes

**DOI:** 10.1038/s41598-017-00833-1

**Published:** 2017-04-13

**Authors:** Ming Ming Yang, Jun Wang, Li Dong, De Ju Kong, Yan Teng, Ping Liu, Jiao Jie Fan, Xu Hui Yu

**Affiliations:** 1 Eye Hospital, the First Affiliated Hospital of Harbin Medical University, Harbin, China; 2 Department of Endocrinology, the First Affiliated Hospital of Harbin Medical University, Harbin, China

## Abstract

Uveitis is a devastating ocular disease that causes blindness. Our previous studies have achieved great advancements in depicting the genetic profiles of uveitis regarding complement pathway genes. This study aimed to provide additional insights into this interest by testing the “central” factor of the complement system, *C3* gene variants, in two uveitis entities. Eight haplotype-tagging SNPs of *C3* gene were genotyped in 141 anterior uveitis (AU), 158 non-infectious intermediate and posterior uveitis (NIPU) and 293 controls. The results showed that none of the tagging SNPs had a significant association with uveitis (*P* > 0.05), either in the global uveitis or subtypes. Although rs428453 showed a nominal association with NIPU subtype in the recessive model (*P* = 0.042), the *P* value could not withstand the Bonferroni correction (*P*_corr_ > 0.05). Stratification analyses according to HLA-B27 status and correlation analysis still did not find any significant interactions or genetic markers regarding AU. Logistic regression analysis also revealed no gender-related epistatic effects of *C3* on uveitis. Two haplotype blocks were defined across the *C3* locus but neither of them was significantly associated with uveitis or subtypes. This study shows no significant association of the *C3* gene with uveitis, suggesting *C3* confers either no or limited risk for uveitis susceptibility.

## Introduction

Uveitis is a group of heterogeneous ocular inflammatory diseases with complex phenotypes, which is considered as a substantial visual impairment as well as an important socio-economic problem, being the fourth cause of blindness worldwide^1, 2^. Uveitis can be frequently classified into anterior uveitis (AU), intermediate uveitis (IU), posterior uveitis (PU), and panuveitis according to the anatomical location of the inflammation^3^. AU, which refers to inflammation of the iris and ciliary body, is the most common form found in clinics. Although less common than AU, non-infectious intermediate and posterior uveitis (NIPU) typically is either idiopathic and comprises many well-defined uveitic ocular conditions or associated with systemic underlying autoimmune disorders, including Vogt–Koyanagi–Harada disease (VKH), Behçet’s disease (BD), and sarcoidosis^3, 4^.

Although the exact pathogenesis of uveitis remains unclear, it is generally accepted as an inflammatory condition and mainly mediated by various endogenous immunological mechanisms^5^. Moreover, genetic factors in the initiation and development of uveitis have been recognized for a few decades^6–11^.

The complement system is a key component of innate immunity and plays an important role in modulating various immune and inflammatory responses. The activation of the complement system occurs along three routes - the classical, alternative, and lectin pathways^12^. Notably, in recent years, accumulating studies have provided increasing evidence that complement is involved in the pathogenesis of uveitis. These studies revealed that activation of the complement system is critical for the development of experimental autoimmune anterior uveitis (EAAU), conversely, depletion of the host’s complement system could result in complete inhibition of EAAU^13, 14^. In addition, several key components and regulators in the complement system have been implicated in the development of uveitis models and other autoimmune diseases. There is a well-established concept that most immune-related disorders share a certain percentage of their genetic component, implying that some pathogenesis may be influenced by common pathways^15^. Evidence from the above studies led us to explore the genetic impact of complement genes on uveitis susceptibility. In our lab, several complement pathway genes have been extensively investigated in two different uveitis entities in our study cohort, AU and NIPU. These genes included complement factor H (*CFH*), complement factor B (*CFB*), complement factor I (*CFI*), Component 1 inhibitor (*C1INH*), and complement component 5 (*C5*)^16–23^.

CFH is a key regulator involved in the complement alternative pathway, and we identified a gender-specific association between AU and *CFH* polymorphisms for the first time. *CFH*-rs1065489 TT genotype was identified as a clinical marker associated with higher uveitis recurrent frequency while interactions with human leukocyte antigens (HLA)-B27 status was also observed^18, 21^. Interestingly, similar associations of *CFH* as described in AU were also found with NIPU patients in our study cohort, suggesting that these two uveitis entities shared general genetic background although presenting different clinical phenotypes^19^. We further demonstrated that genetic variants of *CFB* and *CFI*, in the same complement alternative pathway, are risk factors for both AU and NIPU patients. Moreover, a significant joint-effect among these genes, as well as genotype and phenotype correlations was observed^17, 20, 23^. In addition, parallel studies also demonstrated that genetic variants involved in the alternative pathway, *CFH* and *CFB*, as well as *C5*, considered as the “downstream” complement regulator, were also associated with type 2 diabetic retinopathy (T2DM), viewed from the perspective of inflammation^16, 22^. These results further consolidate the concept that these different immune-mediated diseases may be influenced by common pathways and shared many genetic similarities. Apart from this, we also investigated *C1INH* gene, with a view to elucidating the involvement of the classic pathway in uveitis pathogenesis. The results showed no significant associations with either AU or NIPU patients. Nevertheless, previous reports from our laboratory have successfully established a genetic profile of complement pathway genes in uveitis susceptibility.

Complement component 3 (C3) is the “central” component of the complement cascade and is involved in all three pathways. Genetic deficiency of *C3* has been shown to ameliorate the incidence and severity of EAU^14^. Additionally, variations in the *C3* gene have been associated with several inflammatory diseases, such as age-related macular degeneration (AMD), polypoidal choroidal vasculopathy (PCV), systemic lupus erythematosus (SLE), and inflammatory bowel disease^24–26^. So far however, little is known about the genetic profile of *C3* in uveitis. Together with our previous studies and others, we herein aimed to explore whether *C3* gene variants are involved in the genetic predisposition to uveitis.

## Methods

### Study subjects

The study protocol was approved by Ethnic Committee on Human Research, Harbin Medical University. All the procedures were conducted according to the tenets of the Declaration of Helsinki. Informed consent was obtained from all study subjects after an explanation of the nature of the study.

A total of 299 uveitis patients and 293 control subjects aged ≥55 years without major eye diseases or any systemic immune-related disorders were recruited. The patients were given detailed clinical and ophthalmic assessments, including ocular tonometry, corrected visual acuity, slit-lamp microscopy, and fundus examinations. Clinical information and demographic conditions of the patients were also documented.

The definition of AU was based on the Standardization Uveitis Nomenclature (SUN) classification^27^. All AU patients were recruited during the active phase of uveitis and followed for at least two years after recruitment. The intermediate and posterior uveitis comprise a group of ocular disorders. Considerring that they may share an underlying immune etiology, we combined IU and PU to investigate the genetic impact of complement factors on the whole uveitis susceptibility. Patients were categorized into three specific subtypes: IU, VKH and Behçet’s disease. All IU patients had IU in isolation without posterior uveitis or panuveitis, while VKH and Behçet’s disease patients had either panuveitis or posterior uveitis. Patients with uveitis secondary to ocular or systemic infections were excluded from the study.

### SNP selection and genotyping

Haplotype tagging SNPs across *C3* region were obtained from HapMap Project database for the Han Chinese population (http://hapmap.ncbi.nlm.nih.gov/). Eight SNPs were selected by the tagger-pairwise method with R square and MAF values greater than 0.80 and 0.10 respectively. This set of 8 SNPs captured 96% of alleles in the *C3* locus with MAF larger than 0.1 and a mean *r* ^2^ of 0.97. All SNPs were genotyped by *Taq*Man SNP Genotyping Assays (Applied Biosystems Inc., Foster City, CA) in the Light Cycler 480 Genotyping Master (Roche Diagnostics Inc., Mannhein, Germany) according to manufacturers’ protocols. All PCR amplifications were performed with the following thermal cycling conditions: 95 °C for 10 minutes followed by 40 cycles of 92 °C for 15 seconds, and 62 °C for 1.5 minutes. The HLA-B27 allele was detected by nested polymerase chain reaction (nPCR).

### Statistical analysis

Hardy-Weinberg Equilibrium (HWE) was tested by *χ* ^*2*^ test for genotype frequencies of all SNPs in the control group. Allelic or genotype distribution of each SNP was evaluated using the chi-square test or Fisher exact test (SPSS, version 20.0; SPSS Inc., Chicago, IL). Dominant and recessive models were applied to investigate the disease association with regard to the minor allele. The odds ratio (OR) and corresponding 95% confidence interval (CI) were also estimated. Pairwise linkage disequilibrium (LD, *D’*) and EM-based haplotype association analysis were performed by Haploview (ver. 4.2). Logistic regression analysis was performed to adjust the effect of SNPs with gender. We stratified the study subjects according to subtype, gender and performed association analysis of the SNPs in each gender stratum, *P* < 0.05 was considered statistically significant. *P* values were corrected by Bonferroni test for multiple comparisons (n = total number of SNPs), or permutation test in Haploview software.

## Results

### Clinical Characteristics of Uveitis Entities

In our study, a total of 299 uveitis patients were recruited; which were grouped into AU (141, 47.2%) and NIPU (158, 52.8%) subtypes listed below. NIPU, as a mixed disease entity, is comprised of 51 (32.3%) VKH, 45 (28.5%) IU and 62 (39.2%) BD. The details of clinical subtypes, age, and sex distribution in the uveitis group and controls are shown in Table 1. Gender was generally matched between the AU and NIPU subtypes; whereas, a slight tendency toward a higher proportion of males was observed in the total uveitis group (*P* = 0.097) compared with controls. Therefore, gender was adjusted in the following association analysis by using logistic regression. Among AU patients, 55 (39.0%) were HLA-B27-positive and 86 (61.0%) were HLA-B27-negative.

**Table 1:** Demographic Details of Study Subjects.

	AU (n = 141)	NIPU (n = 158)			Total Uveitis (n = 299)	Control (n = 293)	Comparison	
		VKH (n = 51)	IU (n = 45)	BD (n = 62)			AU *vs*. NIPU	Total *vs*. Control
**Gender (Male/Female)**	72/69	22/29	16/29	42/18	152/147	129/164	0.94	0.097
**Mean age±SD (years)**								
general	50.4 ± 14.6	49.0 ± 16.3	41.3 ± 14.9	49.5 ± 11.2	48.6 ± 14.5	74.3 ± 7.5	0.042	*P* < 0.001
male	50.2 ± 14.4	55.5 ± 16.3	42.3 ± 15.4	50.2 ± 11.6	50.1 ± 14.3	73.7 ± 7.0	0.095	*P* < 0.001
female	50.6 ± 14.9	43.9 ± 14.6	40.8 ± 14.8	47.8 ± 10.5	47.0 ± 14.7	74.8 ± 7.8	0.005	*P* < 0.001
**Age range (years)**								
general	11–87	20–81	18–73	25–69	11–87	55–94	NA	NA
male	18–87	20–81	18–72	25–69	18–87	55–89	NA	NA
female	11–87	23–73	18–73	26–60	11–87	55–94	NA	NA

AU: anterior uveitis; NIPU: non-infectious intermediate and posterior uveitis; VKH: Vogt–Koyanagi–Harada disease;

IU: intermediate uveitis; BD: Behçet’s disease; NA: not applicable;

SD: standard deviation.

The mean age of the AU was significantly greater than that in NIPU patients (*P* = 0.042), which was obvious in the female subgroup (*P* = 0.005). Among NIPU patients, the mean age of VKH was significantly higher than that of IU in general and male subgroups in particular (one-way ANOVA Fisher LSD, *P* = 0.024 and 0.01, respectively). The mean age of the control individuals was significantly greater than that of all uveitis patients as expected (all *P* < 0.001). This is because we purposely recruited subjects older than 55 as control, so as to largely reduce the confounding effects from younger subjects.

### Association analysis

All of the tested SNPs followed HWE in all subjects (*P* > 0.05). The allelic frequencies of all *C3* variants were generally closer, and not significantly different among groups, suggesting that none of the 8 SNPs had allelic association with either global uveitis or its subtypes (Table 2). Also, no SNP showed a significant association with the total uveitis entity or any subtypes in the dominant or recessive genotypic models. Although a trend of higher frequency of rs428453 CC homozygosity was observed in the NIPU subtype compared with that in controls, the difference loses significance after adjustment for multiple testing (*P* = 0.042 and *P*_corr_ = 0.34; Table 3). In the epistatic analysis, no gene*gene interaction was detected between each *C3* SNP and *CFH* rs800292 (I62V) or *CFB* rs1048709 (data not shown). Furthermore, the logistic regression analysis revealed that none of the *C3* variants were significantly associated with uveitis and its two sub-clinical entities after being adjusted for gender and SNP-gender interaction (all *P* > 0.05). In addition, gender independence and SNP-gender effects did not provide further information (Table 4).

**Table 2:** Allelic association of SNPs in C3 genes with AU, NIPU and Total Uveitis.

Variation	Location	Minor allele	Allele Distribution (%)						Allelic Association			
			AU (n = 141)	NIPU (n = 158)	Total Uveitis (n = 299)	Control (n = 293)	AU vs. Control		NIPU vs. Control		Total Uveitis vs. Control	
							*P*	OR(95%CI)	*P*	OR(95%CI)	*P*	OR(95%CI)
rs17030	6628989	G	0.41	0.43	0.42	0.42	0.85	0.97 (0.73–1.30)	0.87	1.02 (0.78–1.45)	0.997	1.0 (0.79–1.26)
rs344555	6630360	A	0.26	0.27	0.27	0.25	0.7	1.07 (0.77–1.48)	0.4	1.14 (0.84–1.56)	0.45	1.11 (0.85–1.44)
rs2241393	6636304	G	0.3	0.36	0.32	0.35	0.27	0.84 (0.62–1.14)	0.55	0.92 (0.69–1.22)	0.31	0.88 (0.69–1.12)
rs2241392	6636983	G	0.33	0.31	0.31	0.32	0.6	1.09 (0.80–1.47)	0.95	0.99 (0.74–1.33)	0.79	1.03 (0.81–1.32)
rs428453	6653157	C	0.16	0.15	0.15	0.16	0.9	0.98 (0.66–1.44)	0.77	0.95 (0.65–1.38)	0.79	0.96 (0.70–1.31)
rs11672613	6656246	G	0.43	0.42	0.42	0.43	0.71	1.06 (0.79–1.41)	0.96	0.99 (0.75–1.31)	0.86	1.02 (0.81–1.29)
rs2230205	6660704	A	0.43	0.42	0.42	0.42	0.79	1.04 (0.78–1.39)	0.89	1.02 (0.77–1.35)	0.81	1.03 (0.82–1.30)
rs2250656	6669534	G	0.24	0.23	0.24	0.24	0.79	1.05 (0.75–1.46)	0.94	0.99 (0.72–1.36)	0.92	1.01 (0.78–1.32)

AU: anterior uveitis; NIPU: non-infectious intermediate and posterior uveitis; C3: complement component 3; CI: confidence interval;

OR: odds ratio.

**Table 3:** Genotypic association of SNPs in C3 genes with AU, NIPU and Total Uveitis.

Variation	Location	Genotype	Genotype Distribution (%)				Genetic model	Genotype Association		
			AU (n = 141)	NIPU (n = 158)	Total Uveitis (n = 299)	Control (n = 293)		AU *vs*. Control	NIPU *vs*. Control	Total Uveitis *vs*. Control
								*P*-value	*P*-value	*P*-value
rs17030	6628989	GG/AG/AA	25/67/49	30/75/53	55/142/102	57/133/103	Dominant	0.94	0.73	0.79
							Recessive	0.67	0.91	0.74
rs344555	6630360	AA/AG/GG	9/55/77	15/56/87	24/111/164	21/105/167	Dominant	0.64	0.69	0.6
							Recessive	0.76	0.38	0.69
rs2241393	6636304	GG/GC/CC	18/50/73	19/64/75	37/114/148	38/128/127	Dominant	0.11	0.44	0.15
							Recessive	0.95	0.77	0.83
rs2241392	6636983	GG/GC/CC	19/55/67	19/60/79	38/115/146	27/132/134	Dominant	0.73	0.39	0.45
							Recessive	0.18	0.35	0.17
rs428453	6653157	CC/CG/GG	5/34/102	9/30/119	14/64/221	5/85/203	Dominant	0.51	0.18	0.21
							Recessive	0.31*	0.042*	0.06
rs116726	6656246	GG/AG/AA	30/62/49	32/68/58	62/130/107	46/158/89	Dominant	0.36	0.17	0.16
							Recessive	0.15	0.22	0.11
rs2230205	6660704	AA/AG/GG	28/64/49	33/67/58	61/131/107	53/142/98	Dominant	0.79	0.49	0.55
							Recessive	0.66	0.47	0.48
rs2250656	6669534	GG/AG/AA	13/43/85	14/46/98	27/89/183	18/105/170	Dominant	0.65	0.41	0.43
							Recessive	0.24	0.28	0.19

AU: anterior uveitis; NIPU: non-infectious intermediate and posterior uveitis; C3: complement component 3; *Fisher exact test.

**Table 4:** Logistic regression analysis of C3 SNPs, gender and SNP*gender interaction.

SNPs	*P* value for SNP effect	*P* value for Gender effect	*P* value for SNP*gender effect
rs17030	0.23	0.75	0.06
rs344555	0.23	0.023	0.14
rs2241393	0.74	0.21	0.68
rs2241392	0.69	0.13	0.81
rs428453	0.28	0.7	0.81
rs11672613	0.17	0.24	0.69
rs2230205	0.52	0.39	0.85
rs2250656	0.051	0.093	0.24

Since HLA-B27 has the strongest association with AU known to date, stratification analysis according to HLA-B27 status was therefore performed. Moreover, correlation analysis with clinical features, such as recurrence frequency, anterior chamber (AC) cells, age of onset, and the presence of posterior synechiae (PS) and keratic precipitates (KP), was also applied. However, we still did not find any associations of *C3* variants with uveitis (Table 5) nor any with clinical features (data not shown). In addition, stratification analysis was performed according to NIPU subtypes (IU and PU), with a view to indentifying specific disease-association. The results did not show any significant associations between *C3* variants and NIPU subtypes, either IU or PU (Table 6).

**Table 5:** Comparison of Allele Frequencies of *C3* Polymorphisms in Patients with AU versus Control Subjects Stratified by HLA-B27 status.

Variation	Minor Allele	HLA-B27 Positive AU (n = 55)	HLA-B27 Negative AU (n = 86)	Controls (n = 293)	*P*-value^§^	*P*-value^£^
rs17030	G	0.42	0.41	0.42	NS	NS
rs344555	A	0.25	0.27	0.25	NS	NS
rs2241393	G	0.27	0.33	0.35	0.26	NS
rs2241392	G	0.31	0.34	0.32	NS	NS
rs428453	C	0.16	0.15	0.16	NS	NS
rs11672613	G	0.32	0.44	0.43	0.16	NS
rs2230205	A	0.37	0.46	0.42	NS	NS
rs2250656	G	0.23	0.28	0.24	NS	NS

Data are the number of subjects (% of the total group); ^§^ *P*-value for HLA-B27-Positive Patients versus Controls; ^£^ *P*-value for HLA-B27-Negative Patients versus Controls; NS Not significant.

**Table 6:** Comparison of genotype and allele frequencies of *C3* polymorphisms in subgroups of Non-infectious Intermediate and Posterior Uveitis.

Variation	Minor allele(%)	IU (n = 45)	PU (n = 113)	Control (n = 293)	IU *vs*. Control	PU *vs*. Control
	Genotype				*P*-value	*P*-value
rs17030	G	35(38.9)	100(44.2)	247(42.2)	0.56	0.59
	GG/AG/AA	6/23/16	24/52/37	57/133/103	0.96^†^ 0.33^‡^	0.65^†^ 0.69^‡^
rs344555	A	20(22.2)	66(29.2)	147(25.1)	0.56	0.59
	AA/AG/GG	3/14/28	12/42/59	21/105/167	0.51^†^ 1.0^‡^*	0.38^†^ 0.25^‡^
rs2241393	G	31(34.5)	71(31.4)	204(34.8)	0.56	0.59
	GG/GC/CC	6/19/20	13/45/55	38/128/127	0.89^†^ 0.95^‡^	0.33^†^ 0.69^‡^
rs2241392	G	29(32.2)	69(30.5)	186(31.7)	0.56	0.59
	GG/GC/CC	4/21/20	15/39/59	27/132/134	0.87^†^ 1.0^‡^*	0.24^†^ 0.23^‡^
rs428453	C	11(12.2)	37(16.4)	95(16.2)	0.56	0.59
	CC/GC/GG	2/7/36	7/23/83	5/85/203	0.14^†^ 0.24^‡^*	0.41^†^ 0.053^‡^*
rs11672613	G	41(45.6)	91(40.3)	250(42.7)	0.56	0.59
	GG/AG/AA	9/23/13	23/45/45	46/158/89	0.84^†^ 0.47^‡^	0.07^†^ 0.26^‡^
rs2230205	A	45(50.0)	88(38.9)	248(42.3)	0.56	0.59
	AA/AG/GG	11/23/11	22/44/47	53/142/98	0.23^†^ 0.31^‡^	0.13^†^ 0.75^‡^
rs2250656	G	17(18.9)	57(25.5)	141(24.1)	0.56	0.59
	GG/AG/AA	3/11/31	11/35/67	18/105/170	0.17^†^ 0.75^‡^	0.82^†^ 0.21^‡^

IU: intermediate uveitis; PU: posterior uveitis, including VKH and Behçet’s disease; C3: complement component 3; Data are the number of subjects (% of the total group) *Fisher exact test; ^†^P-value for dominant model; ^‡^P-value for recessive model.

**Figure 1:**
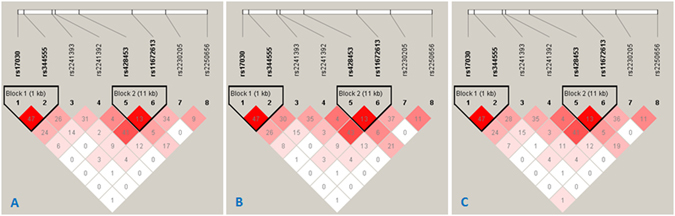
Linkage disequilibrium (LD) structure of the *C3* locus for AU (**A**), NIPU (**B**) and Total Uveitis **(C)** LD was measured using data from all controls, total uveitis and its subtypes. The haplotype block was defined by the confidence interval method implemented in the Haploview software. The LD (*r* ^*2*^) between any two SNPs is listed in the cross cells. AU: anterior uveitis; NIPU: non-infectious intermediate and posterior uveitis.

Pairwise LD analysis was performed across the *C3* locus by using these 8 SNPs, which defined two haplotype blocks in total uveitis entity and its two subtypes (Block 1 involves SNPs rs17030 and rs344555, Block 2 involves SNPs rs428453 and rs11672613; Fig. 1). No haplotype was significantly associated with the diseases (Table 7).

**Table 7:** Haplotype association of *C3* gene with uveitis and its subtypes.

	Frequency				Association (*P*-value)		
	AU	NIPU	Total uveitis	Control	AU *vs*.Control	NIPU *vs*. Control	Total uveitis Control
**Block 1** rs17030-rs344555							
A-G	0.587	0.558	0.579	0.578	0.84	0.62	0.99
G-A	0.245	0.253	0.266	0.251	0.88	0.96	0.55
G-G	0.168	0.189	0.156	0.171	0.94	0.55	0.48
**Block 2** rs428453-rs11672613							
G-G	0.413	0.453	0.424	0.427	0.72	0.30	0.91
G-A	0.418	0.394	0.422	0.411	0.48	0.42	0.69
C-A	0.156	0.152	0.154	0.159	0.69	0.81	0.77

AU: anterior uveitis; NIPU: non-infectious intermediate and posterior uveitis; C3: complement component 3.

## Discussion

Based on the crucial role innate immune mediator C3 plays in EAAU and EAU models, we explored the potential association of the *C3* gene with uveitis. In this study, we identified 8 tag-SNPs from the public database that provided good coverage across the *C3* gene (~43 kb; capturing the majority of common genetic variations in the *C3* locus). To the best of our knowledge, this is the first study examining the association of *C3* polymorphisms in two different clinical uveitis entities but with similar genetic background, AU and NIPU. Our results demonstrated that there was no significant association of the *C3* gene with uveitis.

The complement system belongs to the groups of sensing exogenous and endogenous danger-associated molecular patterns and has been implicated in the pathogenesis of uveitis^14, 28^. The genetic impact of several complement pathway genes on susceptibility to uveitis was extensively investigated. Our results provided novel findings that complement genes play a crucial role in the development of uveitis. Of these, variants in *CFH*, *CFB* and *CFI* involved in the alternative complement pathway, were identified as genetic risk factors for uveitis and specific subtypes. As described above, *CFH*-rs1065489 and *CFB*-rs1048709 were identified as clinical markers associated with a specific disease phenotype. Moreover, joint effects between their risk genotypes conferring a strongly increased risk to uveitis were also found^17–20, 23^. The molecular mechanism might be affecting the binding affinity with C3b and subsequently disturbing the activation of the alternative pathway C3-convertase (C3bBb). C3 is the central component of complement and all the complement activation pathways converge at C3. On activation, C3 can break into C3a and C3b fragment that is called anaphylatoxin, which shows proinflammatory and immunoregulatory actions^29^. Genetic deficiency of C3 has been shown to ameliorate in the incidence and severity of EAU^14^. Additionally, *C3*-deficient mice have been shown to have an impaired ability to produce Th2 cytokines, the latter has been implicated in pathogenesis and regulation of EAU in animals^30, 31^. On the other hand, there are a number of studies showing genetic association of the *C3* gene with multiple inflammatory diseases that share similarities with uveitis^24–26^.

Taking all of the above into account, *C3* could be a good candidate gene for the genetic susceptibility to uveitis. However, in the present study, we failed to identify any significant associations of SNPs or haplotypes with uveitis. Considering the shared genetic component between AU and NIPU identified in our previous studies, both of these subtypes were recruited in the present study, which may help to consolidate our findings and identify more specific associations. In the analysis, multiple comparisons of the allelic and genotype frequency of *C3* variants were performed among different groups: global uveitis patients, AU, NIPU and controls, as well as within NIPU subtypes. The non-significant results identified in this study suggest that the *C3* gene may confer either no or limited risk for uveitis susceptibility, whether in AU or NIPU patients.

Next, several implementation-specific analyses have been performed. Regarding AU, we failed to find any genetic interactions with HLA-B27 status or any clinical markers. Stratification analysis according to NIPU entity was also applied to look for specific disease-association wherein significant associations were not observed between *C3* variants and any subsets, although the sample size within subsets was relatively small. Gender differences in the genetic profiles were reported in uveitis and PCV, in the study of Liu *et al*., *C3* rs17030 showed a male-specific association with PCV, and suggested that the *C3* gene is likely to be a risk factor for the male predominance of the disease^32^. Similarly, our previous studies also found gender may influence the association of complement genes with uveitis^18, 19^. However, no SNP-gender interactions in *C3* were observed in the present study. Further analysis was also performed to determine the linkage of *C3* with other established significant findings, in which no gene*gene interaction was detected. The data strengthen the view that *C3* is unlikely to have a major contribution to uveitis. The genetic alterations in the complement system may be located in “upstream” cascade.

Given the well-established genetic profile of complement genes in uveitis, significant linkage evidence points to the *C3* locus, which encoded a “central” factor of the cascade. However, our results did not show any significant associations of *C3* gene variants with uveitis or any subtypes. Conclusively, this data further enriches our growing understanding of uveitis genetics, and clarifies the specific roles of each complement pathway in ocular inflammatory diseases. Further replication studies are required to clarify the current situation because of the limited samples in this study.

A number of limitations within the current study need to be discussed. First, *C3* is a large gene consisting of 41 exons and containing hundreds of SNPs. We analyzed 8 tag-SNPs to narrow down the regions and aimed to capture the majority of common genetic variations of this gene and they may not sufficiently reflect the disease risk of unexamined variants, as some identified functional SNPs conferring susceptibility to immune-related diseases were not investigated in this study due to less MAF. Second, although we focused extensively on common variants in *C3* itself here, it is possible that variants in other genes important in downstream signaling of the active *C3* fragments may also be significant (i.e., C3aR). It is also possible that epistatic effects of variants in other genes within the complement system (i.e., C5) may contribute further to uveitis susceptibility^33–35^. Last but not least, the results so far failed to identify any significant associations with any subtypes of uveitis due to either a true lack of association or small sample sizes with insufficient statistical power to detect weak associations. Thus, these results should be interpreted cautiously.

In summary, our results demonstrated that common variants in the *C3* gene do not contribute greatly to uveitis susceptibility. The evidence presented so far suggests that genetic and immunologic investigations in uveitis should be focused on the “upstream” process of the complement system. Meanwhile, further studies to identify the rare and causal variants that focus on the possible effects on gene function will further elucidate the role of C3 in ocular inflammatory disorders.
